# Anesthetic Management of Modified Radical Mastectomy in a Patient With Severe Dilated Cardiomyopathy: A Case Report

**DOI:** 10.7759/cureus.105536

**Published:** 2026-03-19

**Authors:** Poonam Saraf, Revanth B Challa, Nand Kishore Joshi, Mangesh Mulaokar, Barkha D Agrawal, Ashutosh Kumar

**Affiliations:** 1 Anaesthesiology, All India Institute of Medical Sciences, Nagpur, Nagpur, IND

**Keywords:** dilated cardiomyopthy, modified radical mastectomy (mrm), regional anesthesiology, supraclavicular brachial plexus block, thoracic epidurals

## Abstract

This case report presents the successful anesthetic management of an 84-year-old woman with severe dilated cardiomyopathy (ejection fraction: 25-30%), atrial fibrillation, and recent angina, undergoing a modified radical mastectomy. Given the risks associated with general anesthesia in this patient due to poor cardiac reserve, a combined regional technique was chosen. Ultrasound-assisted thoracic epidural anesthesia and supraclavicular brachial plexus block were administered, achieving complete anesthesia of the surgical field while maintaining stable hemodynamics throughout the procedure. Careful incremental dosing of bupivacaine supplemented with lignocaine-adrenaline, along with vigilant monitoring, prevented hypotension and arrhythmias. The patient had an uneventful intraoperative and postoperative course and was discharged on the seventh postoperative day. This case highlights the efficacy and safety of dual regional anesthesia in high-risk cardiac patients, reinforcing the role of individualized, ultrasound-guided regional techniques as viable alternatives to general anesthesia in select surgical scenarios.

## Introduction

Dilated cardiomyopathy (DCM) is a form of non-ischemic cardiomyopathy, characterized by reduced contractility caused by a dilated left ventricle or sometimes with biventricular dilatation [1,2]. This results in severe systolic dysfunction, raising the risk of perioperative arrhythmias, heart failure, and sudden cardiac death during the perioperative period [3]. In non-cardiac surgeries, especially involving elderly patients, these cardiac issues significantly increase the risk of hemodynamic instability, leading to increased perioperative complications. The primary perioperative anesthetic goals in patients with DCM include maintaining adequate preload, avoiding myocardial depression, preventing tachycardia, and minimizing sudden changes in afterload to preserve cardiac output. Therefore, careful preoperative optimization and planning are essential in such high-risk patients.

Breast cancer surgeries (e.g., modified radical mastectomy (MRM)) are typically performed under general anesthesia (GA) with regional blocks as adjuncts for analgesia. However, in patients with DCM and a recent history of angina, GA can be particularly hazardous. Induction and tracheal intubation cause sympathetic surges, while anesthetic agents and positive pressure ventilation depress myocardial function and reduce venous return [1,4]. These effects may lead to hypotension, arrhythmias, and even cardiac arrest. Hence, in such cases, it is desirable to avoid GA if an effective alternative exists.

In the literature, regional anesthesia such as thoracic epidural alone or in addition to brachial plexus block has been used as a successful alternative to GA in such high-risk cases [5-8]. It offers the advantage of maintaining spontaneous respiration, thereby entirely avoiding GA, which helps maintain stable hemodynamics, which is critical in such high-risk cardiac patients.

We present the case of an 84-year-old female, American Society of Anesthesiologists (ASA) IV, with severe DCM, a recent history of angina pectoris, and valvular heart disease, who successfully underwent an MRM under a combined ultrasound-assisted thoracic epidural anesthesia (TEA) and ultrasound-guided supraclavicular brachial plexus block, to achieve complete surgical anesthesia while avoiding the cardiovascular stress associated with GA, a combination that has rarely been described in patients with severe cardiac dysfunction undergoing MRM. This report discusses the anesthetic strategy, intraoperative management, and postoperative course, and reviews the relevant literature on the management of high-risk cardiac patients undergoing non-cardiac surgery.

## Case presentation

An 84-year-old woman, weighing 60 kg and classified as ASA IV, was scheduled for a left MRM with axillary lymph node dissection for grade 4 infiltrating ductal carcinoma with mucinous features. The patient was a known case of DCM with a history of recent angina and had a history of previous admissions to the hospital for episodes of congestive cardiac failure. There was no relevant family, surgical, or psychosocial history.

**Figure 1:**
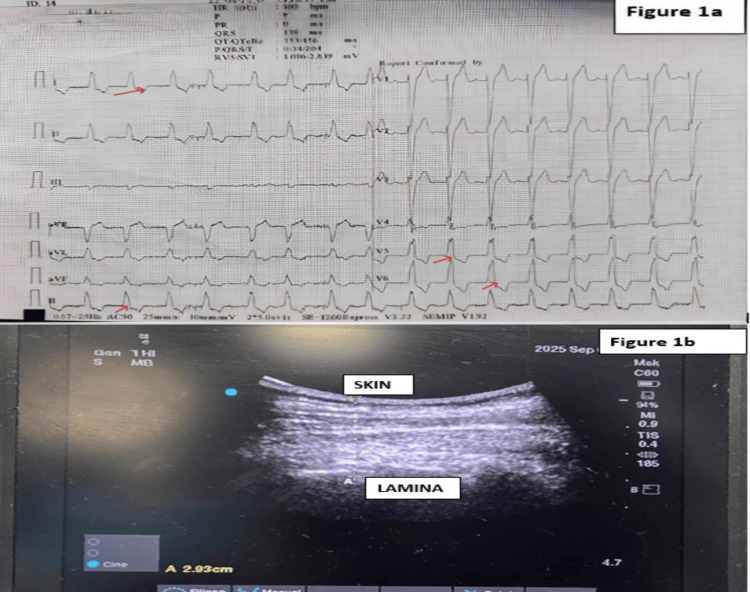
a: Image showing the Left Bundle Branch Block pattern with Atrial Fibrillation in the preoperative ECG. b: Ultrasound Image showing the distance of the lamina from the skin.

On preanesthetic examination, her heart rate was 115 beats/minute and irregular, and her blood pressure was 140/72 mmHg. On auscultation, the chest was clear, and heart sounds were normal. She was able to open her mouth up to 4 cm, and she had a Mallampati class 1 airway. Preoperative ECG showed complete left bundle branch block (LBBB) and atrial fibrillation (Figure 1, Panel a), and the rest of her lab investigations were within normal limits. Two-dimensional echocardiography revealed severe left ventricular (LV) dysfunction, global LV hypokinesia, ejection fraction (EF) of 25-30%, moderate mitral regurgitation, mild pulmonary arterial hypertension, and normal right ventricular function. She was on torsemide 10 mg OD, spironolactone 25 mg OD, ramipril 5 mg OD, and dapagliflozin 10 mg OD for one year.

Considering the high-risk cardiac status and geriatric age group, an ultrasound-assisted thoracic epidural with ultrasound-guided supraclavicular brachial plexus block was decided to be the anesthesia plan. The patient was informed in detail about the procedure and the need for conversion to GA, and high-risk consent was obtained. No premedication was advised.

On the day of surgery, baseline non-invasive blood pressure was 130/86 mmHg, HR was 96 beats/minute, ECG showed LBBB, and oxygen saturation was 97% on room air. A right radial arterial line was placed under local anesthesia. In the sitting position, a 5-1 MHz curvilinear ultrasound probe was used to scan the thoracic vertebrae (Figure 1, Panel b). At the T6-T7 level, the depth of the lamina from the skin was measured at approximately 3 cm. Then, the epidural space was accessed at the T6-T7 level at 5 cm using the loss of resistance to saline technique. The epidural catheter was secured after confirming correct placement with a 3 mL test dose of 2% lignocaine with adrenaline (15 µg). A total of 10 mL of 0.5% bupivacaine mixed with 2 mL of 2% lignocaine with adrenaline was administered incrementally in two aliquots under continuous hemodynamic monitoring. Sensory blockade up to T2 was achieved within 20 minutes. Concurrently, the patient was prepared for an ultrasound-guided supraclavicular brachial plexus block using a 5 cm PNS needle and a 15-6 MHz linear probe. After skin infiltration with 2% lignocaine, 10 mL of 0.25% bupivacaine with 8 mg dexamethasone was deposited around the plexus using an in-plane approach. Within 30 minutes, a satisfactory sensory blockade from T2 to T8 and in the axillary region was confirmed. The total cumulative dose of bupivacaine administered through the epidural and brachial plexus block was 75 mg, which was given in incremental doses under continuous hemodynamic monitoring to ensure safety in view of the patient’s severely compromised cardiac function.

The surgery lasted about 150 minutes. The estimated blood loss was around 400 mL, and the intraoperative course was uneventful with stable hemodynamics and without any need for vasopressors. The patient was shifted to the intensive care unit (ICU) for postoperative monitoring for 24 hours. The arterial line was removed after 24 hours, before the patient’s transfer out of the ICU.

Postoperatively, an epidural top-up for pain management was done with 8 mL of 0.125% epidural ropivacaine every 12 hours along with intravenous analgesics, with the Visual Analog Scale score between 2 and 4. The epidural catheter was removed on the third postoperative day. The postoperative course was uneventful, and the patient was discharged on the seventh postoperative day.

## Discussion

This case highlights the challenges of anesthesia management in a patient with DCM, which is characterized by ventricular dilatation and impaired contractility, which can eventually lead to heart failure. Such patients have a limited cardiac reserve, and even modest shifts in preload, afterload, and heart rate can precipitate acute heart failure, arrhythmias, and hypotension [9]. Moreover, the presence of atrial fibrillation with rapid ventricular response can further compromise diastolic filling and cardiac output. At the same time, a recent history of angina indicates coexistent coronary disease that increases the risk of myocardial ischemia under stress. Indeed, the key goals in DCM include avoiding tachycardia, preventing increases in afterload, and minimizing the negative inotropic effects of anesthetic drugs while preserving the preload [10,11].

GA is associated with considerable risks during induction, as nearly all intravenous and inhalational agents exert a dose-dependent myocardial depression in patients with severe DCM and with a recent history of angina. This can be further worsened by tracheal intubation and positive pressure ventilation. Clearly, in our patient with poor cardiac reserve, a conventional GA approach, even with careful titration and invasive hemodynamic monitoring, carried a substantial risk of intraoperative major adverse cardiac events. This condition provided strong motivation to avoid GA entirely if a suitable alternative anesthetic technique could be employed.

While reviewing the literature, we observed that multiple forms of regional anesthesia, such as TEA, intertransverse process block [12], thoracic paravertebral with pectoralis I block [13,14], and thoracic epidural with interscalene brachial plexus block [15], were used as effective alternatives to GA in high-risk patients undergoing MRM surgery. TEA is known to have multiple benefits, including superior perioperative analgesia compared with systemic opioids, decreased pulmonary complications, shorter mechanical ventilation duration, and shorter postoperative ileus duration after abdominal surgery. One significant advantage of using TEA in patients with DCM is the reduction in afterload, which is beneficial in patients with low EF and protective for perioperative myocardial ischemia [11,16]. Additionally, it blunts the surgical stress response and provides excellent analgesia, thereby preventing tachycardia and hypertension during surgery. By avoiding airway instrumentation and a deeper anesthesia plane, we also minimized the risk of sudden catecholamine surges and arrhythmias. All of these effects were desirable in our patient to prevent myocardial oxygen demand from exceeding supply. In multiple studies and reports, TEA has been seen as a feasible and safer alternative to GA for major breast surgeries, especially in patients with significant comorbidities [5,8,17,18]. Our case adds to this growing experience by showing that even an 84-year-old patient with DCM and an EF of 25-30% can tolerate a major breast surgery under a well-planned regional technique, while maintaining stable hemodynamics throughout the surgery.

MRM with axillary node dissection requires anesthesia of the anterior chest wall from T2-T6 dermatomes as well as the axillary area. Hence, we decided to go with a combined TEA with a supraclavicular brachial plexus block. Using ultrasound guidance for TEA, we could estimate the approximate depth of the epidural space, and in the supraclavicular brachial plexus block, we ensured precise local anesthetic deposition and minimized the volume required, thereby avoiding the complications associated with high-volume brachial plexus blocks. In our case, using 10 mL of 0.25% bupivacaine with ultrasound guidance achieved a complete brachial plexus block without any observable respiratory effects, underscoring the benefit of this careful, image-guided approach.

A critical aspect of our anesthetic strategy was the choice and dosage of local anesthetic to be administered. The severity of physiological effects, such as hypotension, changes in heart rate, and ventricular function, is more pronounced with higher thoracic epidural blocks (above T5) and is directly related to the concentration and volume of local anesthetic used. In our patient, bupivacaine was used for its potent, long-acting analgesia and the dense motor block it provides compared to ropivacaine [19], which is desirable for surgical anesthesia in elderly, high-risk patients. Bupivacaine is known to have a relatively narrow cardiovascular safety margin, and rapid systemic absorption of bupivacaine can cause profound myocardial depression, which can be detrimental in our patient [19]. Hence, by supplementing bupivacaine with 2 mL of 2% lignocaine with 1:200,000 adrenaline, in addition to the test dose of 3 mL of 2% lignocaine with 1:200,000 adrenaline, we could reduce the volume of bupivacaine injected into the epidural space. Faster onset of anesthesia with lignocaine and adrenaline causes vasoconstriction at the injection site, which significantly slows down the vascular uptake of local anesthetic from the epidural space, ensuring a longer duration of surgical anesthesia without the need for top-ups during the surgery. Moreover, we injected our epidural local anesthetic in fractionated aliquots rather than a single bolus, allowing us to reach the desired T2 dermatomal level gradually while closely monitoring the patient’s blood pressure and heart rate, thus avoiding a precipitous sympathetic block.

## Conclusions

This case shows that combining a thoracic epidural and a supraclavicular brachial plexus block can be considered an effective alternative to GA for MRM surgery in patients with severe DCM. Ultrasound guidance, incremental dosing of bupivacaine with lignocaine-adrenaline, and vigilant monitoring minimized hemodynamic compromise and toxicity risk. This highlights the role of dual regional techniques in high-risk cardiac patients and underscores the need for future research into optimal local anesthetic strategies and the broader use of regional anesthesia in similar populations.
